# Reconfigurable Routing Protocol for Free Space Optical Sensor Networks

**DOI:** 10.3390/s120404824

**Published:** 2012-04-13

**Authors:** Rong Xie, Won-Hyuk Yang, Young-Chon Kim

**Affiliations:** 1 Department of Computer Engineering, Chonbuk National University, Jeonju 561-756, Korea; E-Mails: xierong@jbnu.ac.kr (R.X.); whyang@jbnu.ac.kr (W.-H.Y.); 2 Smart Grid Research Center, Jeonju 561-756, Korea

**Keywords:** routing, reconfiguration, free space optical sensor networks (FSOSNs)

## Abstract

Recently, free space optical sensor networks (FSOSNs), which are based on free space optics (FSO) instead of radio frequency (RF), have gained increasing visibility over traditional wireless sensor networks (WSNs) due to their advantages such as larger capacity, higher security, and lower cost. However, the performance of FSOSNs is restricted to the requirement of a direct line-of-sight (LOS) path between a sender and a receiver pair. Once a node dies of energy depletion, the network would probably suffer from a dramatic decrease of connectivity, resulting in a huge loss of data packets. Thus, this paper proposes a reconfigurable routing protocol (RRP) to overcome this problem by dynamically reconfiguring the network virtual topology. The RRP works in three phases: (1) virtual topology construction, (2) routing establishment, and (3) reconfigurable routing. When data transmission begins, the data packets are first routed through the shortest hop paths. Then a reconfiguration is initiated by the node whose residual energy falls below a threshold. Nodes affected by this dying node are classified into two types, namely maintenance nodes and adjustment nodes, and they are reconfigured according to the types. An energy model is designed to evaluate the performance of RRP through OPNET simulation. Our simulation results indicate that the RRP achieves better performance compared with the simple-link protocol and a direct reconfiguration scheme in terms of connectivity, network lifetime, packet delivery ratio and the number of living nodes.

## 1. Introduction

In the past few decades, wireless sensor networks (WSNs), which are composed of numerous nodes randomly distributed over a given region to collect information, have been successfully applied in various applications, such as environmental monitoring, medical care and intelligent households, *etc.* [1]. The sensor nodes form a large scale, multi-hop self-organizing system, communicating their readings to the base station (BS). This type of network has played a significant role in the advancement of ubiquitous computing and mobile communication.

Conventionally, research on WSNs has focused on nodes that transmit data via radio frequency (RF). The signal is transmitted omnidirectionally, so the network is generally modeled as a geometric random graph (GRG). Two nodes, *S_i_* and *S_j_*, can establish a bi-directional link if they are within a fixed communication radius *r*. However, the RF-based sensor networks (RFSNs) suffer from many potential issues, such as severe signal interference, attenuation and collision. As one of the candidate technologies, free space optical sensor networks (FSOSNs) that are based on directed broad-beam free space optics (FSO), show favorable merits over RFSNs. These merits include the increased spatial reuse for communications, smaller node size, ultra-high bandwidths, enhanced security, and the reduction in interference, *etc.* [2].

**Figure 1.**
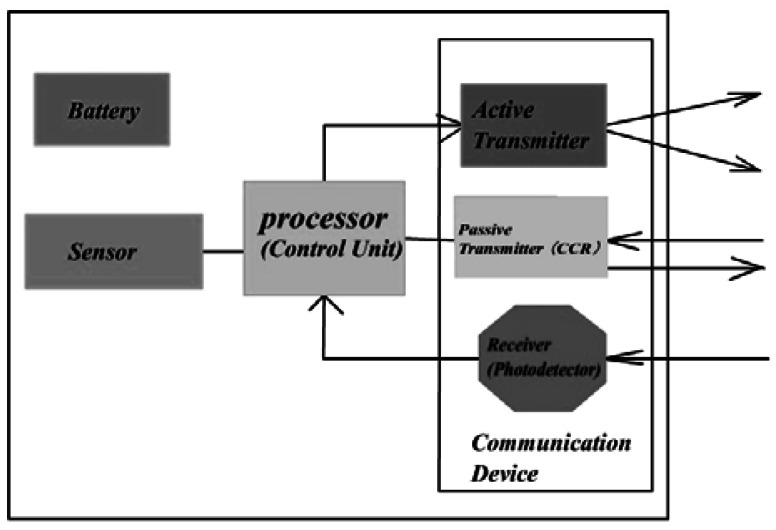
Node architecture.

An increasing number of studies have been focused on FSO-based sensor network systems [3–6]. Just as nodes in RFSNs, nodes in FSOSNs are also generally designed to be small, light and cheap. Typically, a node is equipped with a sensing device (*i.e.*, sensor), tiny battery, simple processor as the control unit, and communication device. What makes nodes in FSOSNs different from those in RFSNs is that their communication device integrates a receiver, an active transmitter, and a passive transmitter based on corner cube retro-reflector (CCR) instead of only a transceiver. Figure 1 shows the general node architecture of FSOSNs. The active transmitter usually assembles a semiconductor laser, collimating lens and beam steering, which is in charge of unidirectional transmission [4]. However, the passive transmitter does not have a light source. Its main component, CCR, consists of three mutually orthogonal mirrors of gold-coated polysilicon that form a concave corner. This CCR has the property that any incident ray of light can be reflected back to the source. Thus, by modulating the reflected beam, it is able to set up a bi-directional link with the light source using a negligible amount of energy consumption [7]. The third part of the communication device is the receiver that is built upon a photo-detector. It can receive signals from most directions.

**Figure 2.**
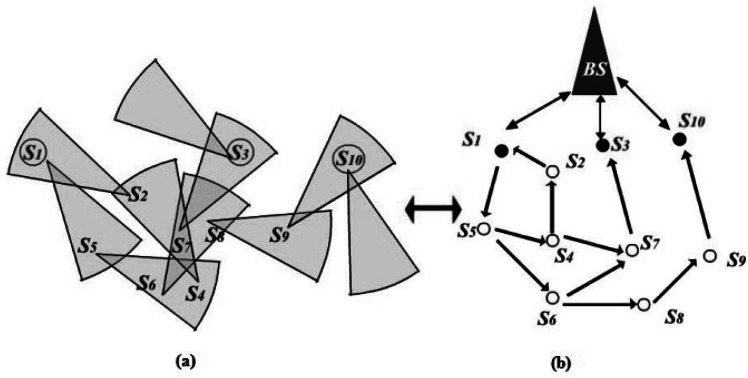
(**a**) An example of FSOSN; (**b**) Hierarchical network structure.

Based on the special node architecture, the establishment of a hierarchical FSOSN is initiated by the BS via scanning the entire area with an interrogating beam of light. Any node in the network that shares a direct line-of-sight (LOS) path with the BS can set up bi-directional path with it using CCR. These nodes are called cluster heads (CHs). All other nodes, known as general sensors (GSs), have to contact the BS through one or multi-hops to the closest CH using active transmitters. Figure 2(a) shows an example of FSOSN, and Figure 2(b) is the corresponding hierarchical network structure. Note that *S_1_*, *S_3_*, *S_10_* are CHs while other nodes are GSs. It is observed that the CHs form the middle layer that acts as a bridge between the BS and GSs with fare energy consumption: the passive transmitter is used when contacting the BS while the active transmitter is applied when contacting the GSs.

**Figure 3.**
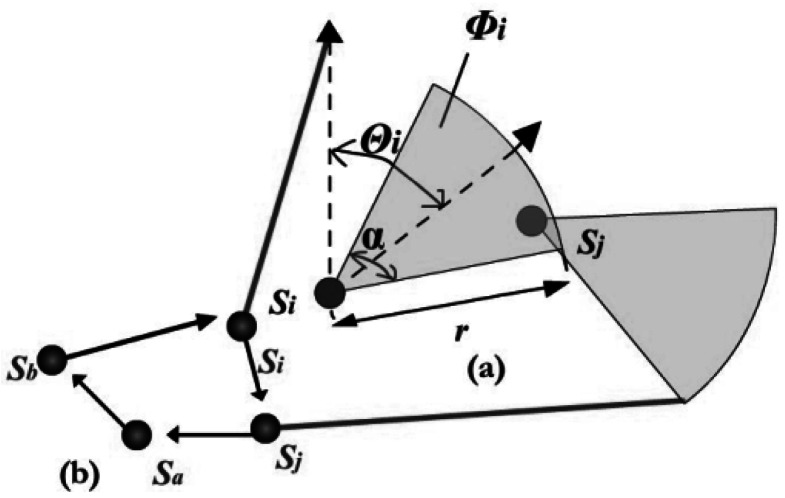
(**a**) Network parameter; (**b**) *S_j_* can talk to *S_i_* through multiple hops *S_j_*→*S_a_*→*S_b_*→*S_j_*.

Compared with the RFSNs, which use omnidirectional communication, the main limitation of FSOSNs is the requirement of a LOS path between a sender and a receiver. With an active transmitter, a node in the FSOSNs transmits data by scanning a directional modulated visible or infrared (IR) beam over a “pie-shaped” angular sector. The network thus has been modeled as a random-scaled sector graph (RSSG) composed of a set *S_n_* = {*S_i_*|*i* = 1, 2, …, *n*} of *n* sensor nodes that are randomly distributed in a given area [8]. Each node with random position (*x_i_*, *y_i_*) has a given communication radius *r* and a random direction *θ_i_*. It can orient the active transmitting beam within a scanning area that covers a contiguous sector of *α* rad. This angle of the scanning area is called the scanning angle and its range is $\left[-\frac{\pi }{2}+\theta_{i},\frac{\pi }{2}+\theta_{i}\right]$. Figure 3(a) shows the network parameters. Note that *S_i_* and *S_j_* are extracted from the network example given by Figure 3(b). For node *S_j_* to hear *S_i_*, we must have that: *d* (*S_i_*, *S_j_*) ≤ *r*, and (*x_j_*, *y_j_*) ∈ *Φ_i_*, where *d* (*S_i_*, *S_j_*) is the Euclidean distance between nodes *S_i_* and *S_j_*, and *Φ_i_* is the scanning area of node *S_i_*. Two distinct sets of neighbors are defined for each node *S_i_*: the set *FNeb*(*S_i_*), known as *S_i_*'s forward neighborhood, contains all nodes that *S_i_* can talk to directly; and the set *BNeb*(*S_i_*), known as *S_i_*'s backward neighborhood, includes all nodes that can directly talk to *S_i_*. In Figure 3(b), *S_j_* is a successor of *S_i_* and *S_i_* is a predecessor of *S_j_* [9]. *S_i_* can get to *S_j_* by one hop transmission, but *S_j_* has to visit *S_a_* and *S_b_* before reaching to *S_i_*.

The design of algorithms and protocols for FSOSNs is very challenging. First, though current technology continues to drive the advances in sensor fabrication, including processing design and computing, advances of battery technology still lag behind, making energy resources the fundamental constraint in sensor networks, whether in RFSNs or FSOSNs. Besides, because of the large amount of nodes, recharging the battery when exhausted is unpractical for sensor network applications, particularly for those operating in hostile environments like volcanoes and swarms. Moreover, especially for FSOSNs, the property of directionality demands high network connectivity for data communication. For example, as the time goes by during data transmission, when a certain sensor node died of the depletion of energy, the network connectivity would probably drop dramatically, because the BS would lose contact with those nodes that had to go through the dead one for up-link communication. Any data originated from these nodes was meaningless and wasting energy. As a consequence, power and connectivity management becomes a main ingredient in the design of algorithms and protocols.

A recent trend in wireless directional communication aims at leveraging the smart antenna, which either consists of *N* beam patterns or can dynamically change the direction of the beam [10,11]. Inspired by this technology, we assume that the sensor nodes in our work are able to reconfigure their direction of the active transmitter during data transmission. Accompanied with the leverage of localization techniques such as global positioning system (GPS) [12], in this paper, we propose a reconfigurable routing protocol (RRP) for FSOSNs to achieve efficient data delivery and extended lifetime by network reconfiguration. Specifically, RRP focuses on dynamically adjusting the orientation and communication radius of nodes during data transmission so as to maintain the network connectivity, guarantee packet delivery, and prolong lifetime. It works in three phases: (1) virtual topology construction, (2) routing establishment, and (3) reconfigurable routing. The first phase acquires global information for the BS, preparing necessary conditions for finding the shortest hop paths from each node to the BS in second phase. During reconfigurable routing, the data is first routed through the shortest hop paths. Then, a reconfiguration is initiated by the node whose residual energy falls below a threshold. An SOS message is sent by this dying node to the BS for further processing. Upon receipt, the BS classifies those nodes affected by the dying one into different types, and reconfigures them respectively by replying messages of reconfiguration. This instant interaction between the BS and sensor nodes allows effective adjustment of the network virtual topology, which prevents the dying node from jeopardizing the data transmission of other nodes and contributes to the network performance.

The rest of this paper is organized as follows: Section 2 presents related works, while Section 3 introduces the proposed RRP in detail. Section 4 explains the simulations experiments. Section 5 provides insights into the analysis of the simulation results. Section 6 discusses the impact of message exchange on energy consumption during reconfiguration. Finally, the conclusions are presented in Section 7.

## 2. Related Works

**Figure 4.**
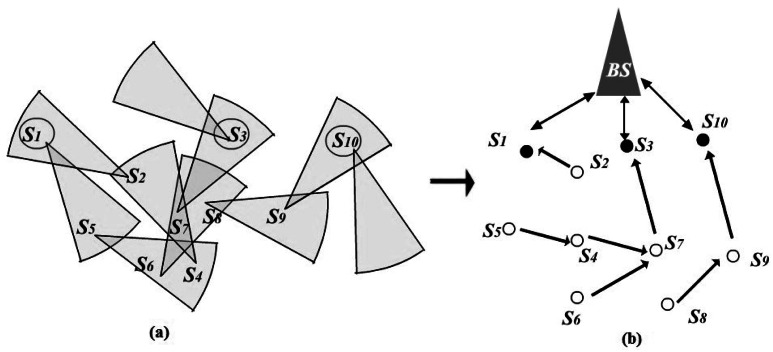
(**a**) An example of FSOSN; (**b**) Virtual topology after running the simple-link protocol.

The directionality of FSOSNs demands efficient routing protocols for network communication. There have been several works focused on routing in FSOSNs. In [8], Diaz *et al.* proposed two protocols, namely simple-bro and simple-link. Simple-bro is a classical flooding based protocol that enables communication from the BS to sensor nodes. Correspondently, the simple-link protocol aims to establish a route from each sensor node to the BS by assigning each node an uplink node to orient. Consequently, the computed set of routes from sensor nodes to the BS forms an oriented forest with roots in the CHs. Figure 4(a) is the same example as Figure 2(a), and Figure 4(b) shows its corresponding network virtual topology after running the simple-link protocol. Obviously, this virtual topology is fragile when facing the death of nodes. That is because the death of a parent node in one tree would probably result in a tremendous amount of children nodes being separated from the network, and thus degrading the network communication. In Figure 4, when *S_7_* died, *S_4_*, *S_5_*, and *S_6_* would totally lose the communication with the BS, even if they still kept sufficient energy.

In [9], two circuit-based algorithms, known as neighborhood discovery algorithm (NDA) and base station discovery algorithm (BDA), were introduced by Okorafor *et al.* NDA attempts to acquire the local neighborhood by finding one most efficient circuit from a node through each of its successors. The destination of each circuit is the originating node itself. And the found circuit also represents the best routes for each of the nodes along that circuit. The NDA provides a significant reference for local neighborhood discovery and maintenance in directional networks. Our previous work in [13] has evaluated the performance of NDA affected by directionality. Another circuit-based algorithm, the BDA, enables BS to get the global network information by flooding the cluster route packets (CRPs). The global information reflecting the network topology is stored in the base stations' routing table (BSRT). This table is useful in constructing routing paths in the initial route discovery, and conducting affected nodes of alternative paths for route maintenance in the case of link failure. However, the details of the routing and maintenance strategies are not discussed in this paper, which is still an open research topic.

Exploiting the security benefits of link directionality, Okorafor [14] introduced a novel light-weight circuit-based, secure and integrated routing and localization paradigm within the FSOSNs. This scheme leverages the hierarchical cluster-based organization of the network, and the directionality of the links to deliver enhanced security performance including per hop and broadcast authentication, confidentiality, integrity and freshness of routing signals. Although the performance of this scheme was evaluated through comprehensive probability and simulation analysis on security, the author did not discuss or evaluate the performance in terms of energy conservation, which is of great significance in sensor networks.

Actually, to guarantee efficient data communication in FSOSNs, a routing protocol with dynamic topology reconfiguration is crucial for two reasons. First, with reconfiguration, the network connectivity can be better maintained despite the constraint of directionality, which facilitates the delivery of data packets. Besides, energy expenditure can be managed more effectively, because the intensive strain of invalid transmission resulted from the lack of connectivity will be released through network reconfiguration. Therefore, RRP is proposed in this paper. To the best of our knowledge, this is the first work of reconfiguring sensor nodes in FSOSNs, the details of which are shown in the following section.

## 3. RRP Description

The RRP works in three phases: (1) virtual topology construction, (2) routing establishment, and (3) reconfigurable routing. The first phase acquires the global network information for BS, which prepares the necessary condition to construct the initial routing paths, typically the shortest paths, for all sensor nodes in the second phase. Reconfigurable routing is a working phase in which the sensor network starts its task with a dynamic reconfiguration. The following sections explain each phase in details.

### 3.1. Virtual Topology Construction

**Figure 5.**
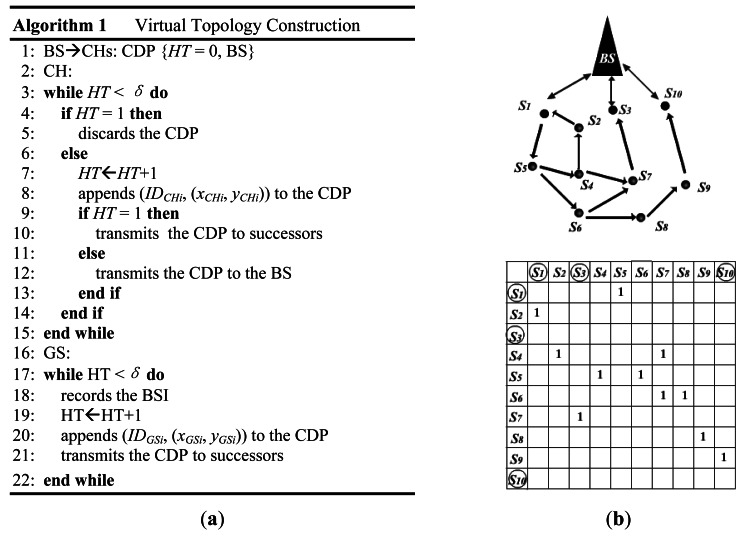
(**a**) Pseudo-code of virtual topology construction; (**b**) an example of network virtual topology, which results from the FSOSN in Figure 2 after the 1st phase of RRP.

Virtual topology construction takes place right after the deployment of all sensor nodes. It is completed by flooding a circuit discovery packet (CDP) initiated from the BS. The process is similar to BDA in [9], but we use CDP in our protocol. A CDP contains a hops-traversed (HT) field and a BS-information (BSI) field in its header. The HT counts the number of hops it has traversed and the BSI records the BS location information. When a CH receives a CDP, it has to verify this CDP by checking the HT. If HT = 1, then the CDP does not travel through the GSs and contains no useful information. Such CDP will be discarded by the CH. Otherwise, the CH increases the HT by one, and appends its own information including the ID and location to CDP. Then, it forwards the CDP to its successors in the downlink case (HT = 1), or to the BS in the uplink case (HT > 1). Note that in the case of a downlink, the CDP arriving at CHs has not yet traveled the GSs layer, so the HT filed equals to 1 when the CDP is forwarded; but the situation is the opposite in an uplink case. When a GS receives a CDP, it records the BSI contained in the packet, increases the HT by one and then forwards the CDP to its successors. The CDP is considered to be expired if the HT is larger than a given constant *δ*. When a CDP returns to BS, the BS gets the nodes' location and extracts the path that is formed by the ID sequence in the CDP to create entries into a GlobalGraph (*n* × *n*). Figure 5 shows the pseudo-code and the GlobalGraph of the FSOSN in Figure 2 after this phase.

### 3.2. Routing Establishment

This phase constructs the initial routing paths for each sensor node in the network. The shortest hop paths are used, considering the minimization of delay and energy consumption. After the first phase, the BS is able to extract all the shortest hop paths from GlobalGraph (*n* × *n*). With a panorama of the network, it then constructs and updates the routing table of each node.

There are three steps in this phase: (1) uplink graph construction, (2) routing table construction, and (3) routing table update. The uplink graph, denoted by UplinkGraph (*n* × *n*), contains all of the shortest hop paths from sensor nodes to the BS. When the uplink graph is obtained in the first step, the BS computes the routing table (RT) for each node using this graph. There are two fields included in a GS's RT: the next hop set and cost. The next hop set records the IDs of the next hop candidates for the uplink communication of the current node, and the cost is the hop count from the current node to the BS. After extracting the routing table of each sensor node in the uplink graph, the BS unicasts the computed RT to each node with the knowledge of the node's location in the third step for data transmission. Upon receipt, each sensor node stores the RT for communication.

**Figure 6.**
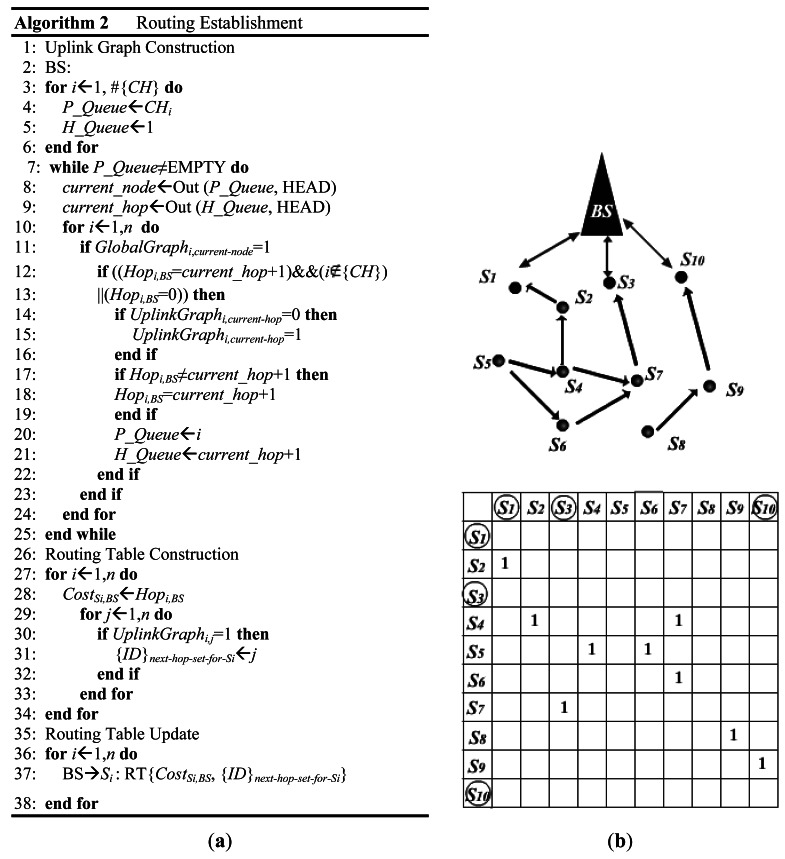
(**a**) Pseudo-code of routing establishment; (**b**) an example of uplink graph, which results from the FSOSN in Figure 2 after the 1st phase of RRP.

The pseudo-code of this phase is shown in Figure 6(a). The uplink graph construction is similar to the level first search (LFS) algorithm. Two queues, *P_Queue* and *H_Queue*, are used in pairs. *P_Queue* is for queuing the sensor nodes from low level to high level, and *H_Queue* is the hop count between the corresponding sensor node in *P_Queue* and the BS. Note that CHs are in the lowest level, because it takes them only one hop to get to the BS. The BS maintains a *Hop* value, which is initially set to 0, for each node. This *Hop* value records the smallest number of hops from the sensor node to the BS. Figure 6(b) gives an example of the uplink graph, which results from the FSOSN presented in Figure 2 after running this routing establishment phase. Table 1 outlines the results of the RT of each GS.

### 3.3. Reconfigurable Routing

The network starts its task with data transmission in this phase. Initially, each node forwards the sensed data to the BS by randomly selecting an ID from its next hop set in the RT. As time goes by, if the residual energy of a node reaches a threshold, it immediately sends an SOS message to the BS for reconfiguration. The SOS message includes two fields: message type and the ID of the dying node. The threshold, denoted by *E_threshold_* is set by the following equation:

(1) $$E_{\text{threshold}}=p\times E_{\text{init}}$$

where *E_init_* is the initial energy of a node and *p* is a percentile that can be appropriately set by the network designer according to specific traffic load and application.

When the BS receives this SOS message, it begins reconfiguring the network by first figuring out those nodes affected by the dying node (DN). The affected nodes can be classified into two categories: maintenance nodes (MNs) and adjustment nodes (ANs). MNs are those nodes whose reconfiguration can be done by modifying their RT. However, for the ANs, adjusting the orientation and communication radius is needed for their reconfiguration. Both MNs and ANs are the predecessors of the DN. The difference is that the MNs can contact the BS through other nodes except the DN. However, the DN is the only choice for ANs to communicate with the BS. Taking the example of Figure 6, *S_4_* is a MN and *S_6_* is an AN when *S_7_* is dying.

After the classification, the BS reconfigures the DN, MNs and ANs as follows:

For the DN, the BS sends it a death notification message directly. This message contains the type of reconfiguration and the ID of the DN. Then the DN establishes a bi-directional path with the BS. If the DN is a CH, then the bi-directional path has already been set up by its CCR; otherwise, if the DN is a GS, then this can be done by re-orienting the DN's active transmitter and adjusting its communication radius to reach the BS. Finally, the DN is isolated from others, and it directly transmits the packets to the BS.

For MNs, the BS sends each of them a message for routing maintenance, as the path to the DN becomes invalid. This message includes three fields: message type, ID of the intended MN, and maintenance information. The message type tells the node this is a message for routing maintenance. The maintenance information specifies the ID of the DN. Upon receipt, each MN eliminates the ID of the DN in the next hop set of its RT to exclude this path.

For ANs, the adjustment of orientation and communication radius is essential because the DN is their only successor for uplink communication. To depict this scheme, some useful denotations are introduced in Table 2. Note that we define a circle area for each AN as *A_AN_*, which is the circle centrally located at the AN with a radius of communication range. When a GS satisfies the connectivity requirement, this means there is at least one path available for it to reach the BS.

**Figure 7.**
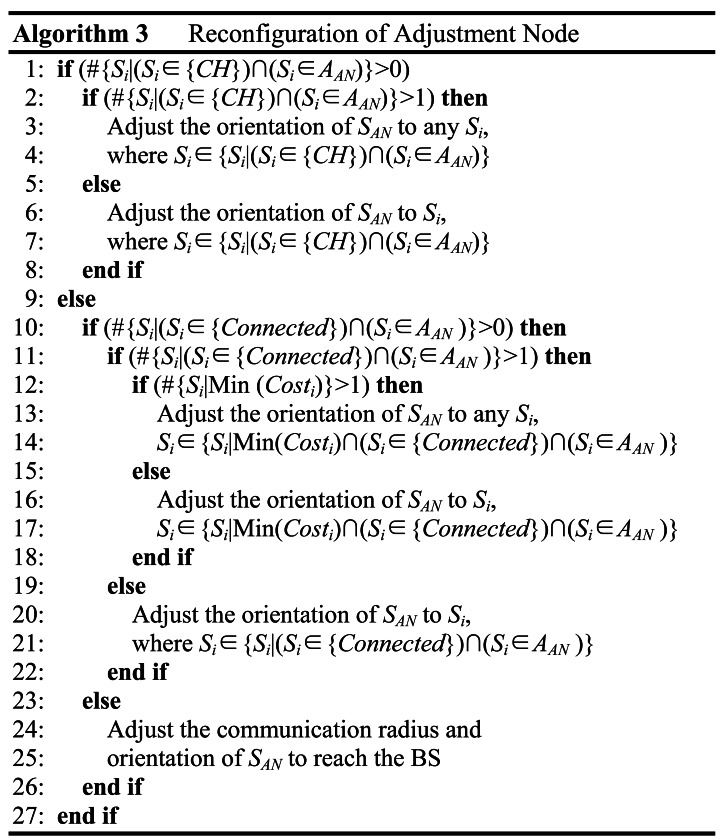
Reconfiguration of adjustment node.

Figure 7 shows the pseudo-code of reconfiguring the AN. To minimize energy consumption, at first, the BS tries to find a living CH in the AN's circle area *A_AN_*. If such a CH exists, then the orientation of AN should be adjusted to the direction of the found CH. If there are more than one qualified CHs, then one of them is chosen randomly. Otherwise, if no such CH is available, then the BS turns to find a GS that satisfies the connectivity requirement of the reconfiguration of AN. If there are two or more candidates, then the one with the smallest number of hops to the BS is selected. However, if none of the qualified CH or GS is found, then the BS regards this AN as an uplink cluster head (UCH) whose orientation should be fixed directly to BS itself for relaying data. When the plan of reconfiguration is ready, the BS directly sends the AN a message including information of the message type, ID of the intended AN, ID of the DN, the adjusted orientation, communication radius, and cost. Upon receipt, the AN does the corresponding adjustment of its orientation, communication radius as well as the RT to complete the reconfiguration.

## 4. Simulations

Our simulations are conducted through OPNET Modeler 11.5. In this section, an energy model is first introduced, followed by a description of protocols for comparison and the simulation scenario.

### 4.1. Energy Model

A different assumption of node characteristics, including energy consumption of the transmitter and receiver, will affect the advantages of different protocols. In our simulations, we apply the same node architecture as shown in Figure 1. According to this architecture, the total energy required for transmitting (*E_TX_*) includes the energy consumed by the active transmitter (*E_AX_*) and that consumed by the passive transmitter (*E_PX_*):

(2) $$E_{TX}=E_{AX}(m,d,\varphi )+E_{PX}(n)$$

For simplicity, we assume that energy loss is proportional to the scanning area when using the active transmitter. Thus, power at the active transmitter should be appropriately adjusted to invert this loss so as to ensure a certain power at the receiver. During data transmission, if the number of bits transmitted by the active transmitter is *m*, then the scanning angle is *φ*, the communication radius is *d*, and then the energy for the active transmitter is:

(3) $$E_{AX}(m,d,\varphi )=ɛ\times \varphi \times d^{2}\times m$$

For the passive transmitter, if the total number of bits transmitted by the CCR is *n*, and the transmitted energy per bit for the CCR is *E_CCR_*, then the energy of passive transmitter is:

(4) $$E_{PX}(n)=E_{\text{CCR}}\times n$$

In the receiver, if the received energy per bit is *E_R_*, then the total energy for receiving *k* bits is:

(5) $$E_{RX}(k)=E_{R}\times k$$

Researchers have designed several energy saving sensor network systems so far. In [4], the estimated energy for *E_CCR_* and *E_R_* is 16 pJ/bit and 69 pJ/bit, respectively. In our simulation, we use the value 16 pJ/bit for *E_CCR_* and set the *E_R_* as 60 pJ/bit for simplicity. The parameter *ε* relies on the required receiver sensitivity and the receiver noise figure as the transmit power needs to be adjusted so that the power at the receiver is above a certain threshold, *P_r-thresh_*. We work backwards to determine the minimum transmit power. If the radio bitrate is *R_b_*, then the transmit power in 1 rad *P_t_* is equal to the transmit energy per bit *E_AX_*(1, d, 1) times the bitrate:

(6) $$P_{t}=E_{AX}(1,d,1)\times R_{b}=ɛd^{2}R_{b}$$

The free space model is adopted in our simulation. According to the Friss free space equation, the transmit power is attenuated as follows:

(7) $$P_{r}(d)=\frac{P_{t}G_{t}G_{r}\lambda^{2}}{{\left(4\pi d\right)}^{2}L}$$

where *P_r_*(*d*) is the receive power given a transmitter-receiver separation of *d*, *P_t_* is the transmit power, *G_t_* is the gain of the transmitting antenna, *G_r_* is the gain of the receiving antenna, *λ* is the wavelength of the carrier signal, *d* is the distance between the transmitter and receiver, and *L* ≥ 1 is the system loss factor not related to propagation. Combining Equations (6) and (7), we get:

(8) $$P_{r}=\frac{ɛR_{b}G_{t}G_{r}\lambda^{2}}{{\left(4\pi \right)}^{2}}$$

Thus, *ε* can be determined by setting Equation (8) equal to *P_r-thresh_*:

(9) $$ɛ=\frac{P_{r-\text{thresh}}{\left(4\pi \right)}^{2}}{R_{b}G_{t}G_{r}\lambda^{2}}$$

We employ the receiver threshold *P_r-thresh_* ≥ −52 dBm used in [15], which could achieve a minimum of 30dB signal-to-noise (SNR) to receive the signal with no error. The whole system works with the following parameters: *G_t_* = *G_r_* = 1, no system loss (*L* = 1), *R_b_* = 250 Mbps, 300 GHz radios and $\lambda =\frac{3.0\times 10^{8}}{300\times 10^{9}}=0.001\;\text{m}$. Then, *ε* = 4 pJ/m^2^ is determined. The parameters in our energy model are summarized in Table 3.

### 4.2. Protocols for Comparison

The performance of our proposed protocol is compared with the simple-link protocol and a direct reconfiguration scheme (DRS). The simple-link sets up the communication between BS and GSs by allocating each GS an uplink node that is one hop closer to the BS. The details of this protocol are introduced in [8], and it works without any reconfiguration scheme. Eventually, the computed set of paths from GSs to the BS forms an oriented forest with the roots in the CHs. A DRS is also designed to evaluate the advantages of RRP, especially the reconfiguration routing in RRP. The DRS is a simple routing strategy, which has two phases: routing establishment and data transmission.

- Routing Establishment
  - The BS broadcasts a packet with its location information to CHs.
  - Upon receipt, each CH checks if its flag *rv_bs_info* initially set as *false* has been changed to *true*. If yes, then it discards the received packet. Otherwise, it stores the information of BS and then marks the flag *rv_bs_info* as *true*, indicating that it has learnt the BS information. Afterwards, the CH broadcasts the packet to its successors.
  - When a GS gets a packet, it also checks the flag *rv_bs_info*. The packet is discarded if the flag is *true*. Otherwise, the GS stores the information of BS and then marks the flag *rv_bs_info* as *true* before broadcasting it to the successors.
  - After the broadcasting period, each GS has learnt the BS information and then adjusts its orientation and communication radius to reach the BS for data communication.
- Data Transmission
  - The CHs transmit the sensed data to the BS by their passive transmitter. The GSs, which have set up direct communication with the BS in the former phase, transmit their sensed data by their active transmitter.

### 4.3. Simulation Scenario

**Figure 8.**
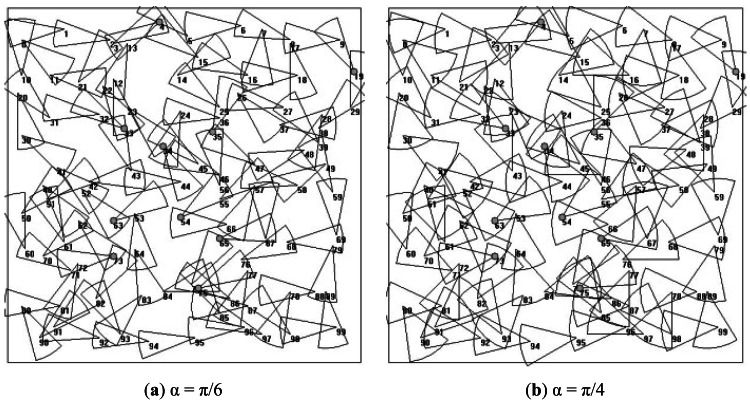

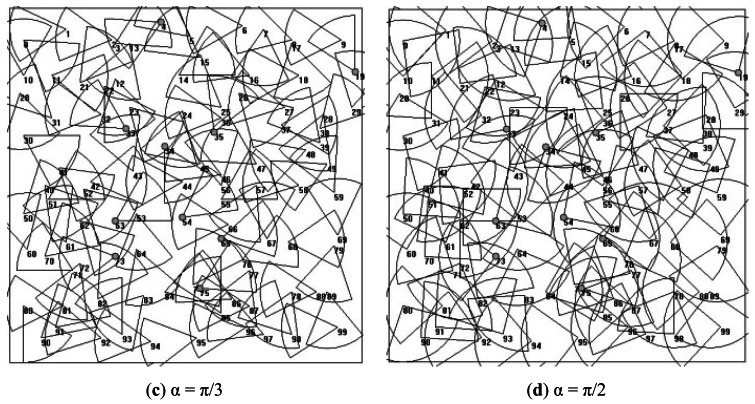
Example of simulated topologies.

We consider a rectangular area of 100 × 100 m^2^ in which 100 sensor nodes are deployed with network connectivity. It means that first each sensor node has at least one path to reach the BS. 10% of the nodes are chosen randomly as CHs. A simple scenario in monitoring application is simulated, which is that each sensor node transmits its sensed data to the BS every 25 s. The length of data packet is fixed at 2,048 bits and the initial energy of each node is 10^−3^ J. To examine the performance influenced by the scanning angle, which is the main factor of directional communication, the scanning angle is set to $\frac{\pi }{6}$, $\frac{\pi }{4}$, $\frac{\pi }{3}$, $\frac{\pi }{2}$, respectively. Ten different topologies are generated and the results are based on the average. An example of the simulated topology is shown in Figure 8.

## 5. Results and Analysis

The performance of the proposed protocol is evaluated in terms of connectivity, network lifetime, packet delivery ratio, and number of living nodes.

### 5.1. Connectivity

Connectivity (CN) is defined as the ratio of the number of nodes involved in valid uplink communication (*N_C_*) to the total number of nodes (*N_T_*) in the network, shown as Equation (10). Note that a node involved in valid uplink communication means that it has at least one path to reach the BS:

(10) $$CN=\frac{N_{C}}{N_{T}}$$

**Figure 9.**
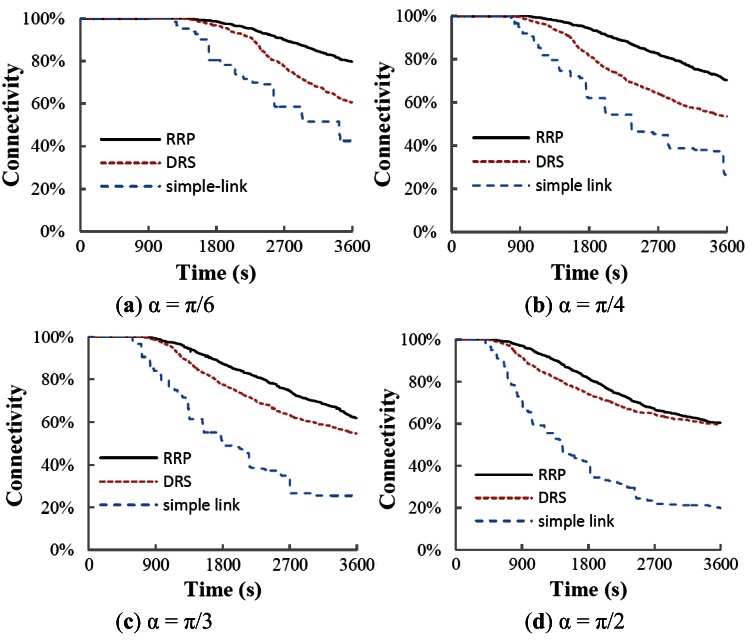
Connectivity *vs.* time under different scanning angles.

Figure 9 shows the results of connectivity versus time of three protocols. RRP outperforms the simple-link protocol and DRS under each simulated scanning angle. Simple-link tries to construct an uplink forest for data communication, so each node in the network has and only has one parent to forward sensed data. This protocol works without any reconfiguration scheme. Once a node runs out of energy as time goes by, it is expected that the network has a dramatic drop of connectivity since the dead node will cause its entire offspring to lose their communication with the BS. However, in DRS, nodes are isolated from each other after the phase of routing establishment. Similarly, in RRP, the dying nodes are also separated from other nodes when a SOS message has been sent out. The death of a node in both DRS and RRP does not influence the uplink communication of other nodes. Therefore, the connectivity of both DRS and RRP presents a continuous dropping trend while the simple-link shows a discrete dropping trend.

**Figure 10.**
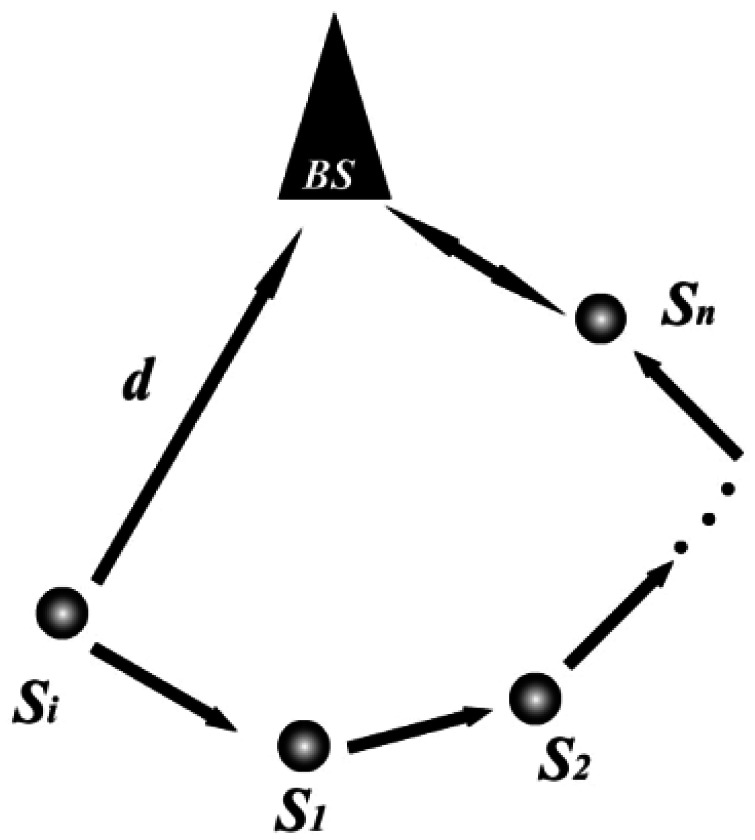
Comparison of direct and multi-hops communication.

Using DRS, it is observed that the network forms a star topology with the BS at the center after the phase of routing establishment. Sensor nodes use direct communication with the BS, which is different from RRP that includes both multi-hops and direct communication. To illustrate the difference of energy dissipated in DRS and RRP, we consider the energy expended in transmitting a single *k*-bit message from a GS, say *S_i_*, located a distance *d* from the BS (shown in Figure 10) using a direct communication approach, then we have:

(11) $$E_{\text{direct}}=k\times ɛ\times \varphi \times d^{2}$$

The energy required for multi-hops routing is:

(12) $$E_{\text{multi}-\text{hops}}=nE_{AX}(k,r,\varphi )+nE_{RX}(k)+E_{PX}(k)$$

Then, the direct communication requires less energy than multi-hop communication if *E_direct_* < *E_multi_*_−_*_hops_*. According to Equations (3–5), we have:

(13) $$d<\sqrt{n(r^{2}+\frac{E_{R}}{ɛ\times \varphi })+\frac{E_{\text{CCR}}}{ɛ\times \varphi }}$$

Equation (13) implies that whether or not direct communication can reserve more energy than multi-hop communication closely relies on *d* and *n* when the network is set up. In DRS, the further a node is away from the BS, the faster it consumes energy. Those nodes located around the bound area generally die quickly. On the contrary, in RRP, most nodes in the network are just a few hops away from the CH, which can slow down their energy consumption rate. Besides, if a node becomes a DN, then its ANs will seek another short path back to the BS by reconfiguration. They will be oriented with the BS if and only if no qualified candidate in their circle area is available. Such strategy reduces the probability of direct transmission that may consume more energy if Equation (13) is not satisfied.

Interestingly, the advantage of RRP over DRS in connectivity tends to get smaller as the scanning angle increases. Though in the case of larger scanning angle, a node may have higher probability to find a shorter path to the BS, the energy needed per transmission also increases. Actually, in our simulation scenario, which has sparse node density, the expanded scanning angle has speeded up the energy consumption as well as the start of reconfiguration in RRP. A node is likely to have lower probability to find a qualified candidate in its circle area, and in turn it uses direct communication with the BS, removing the gap between DRS and RRP.

### 5.2. Network Lifetime

**Figure 11.**
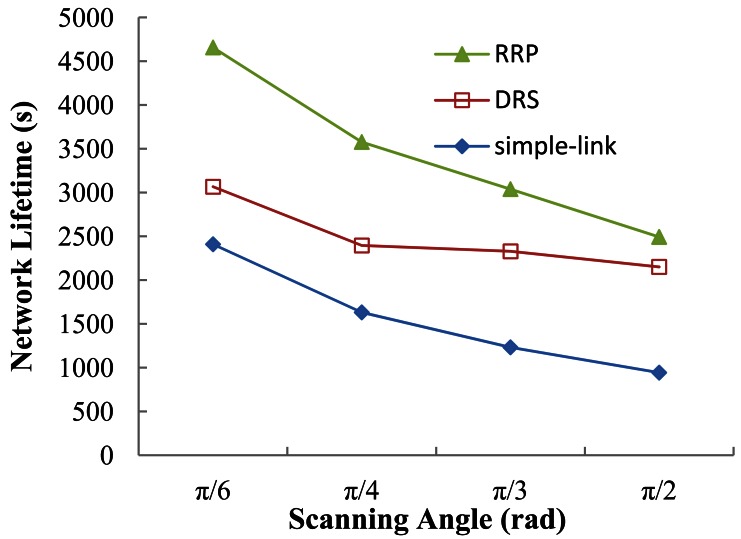
Network lifetime *vs.* scanning angle.

Different definitions of network lifetime exit. In this paper, we define the network lifetime as the period from when the nodes begin to transmit sensed data till the time when the network connectivity drops to 70%, considering that the lack of connectivity may cause severely ineffective transmission. Figure 11 shows the results of network lifetime versus different scanning angles. Obviously, the performance of RRP and DRS is superior to that of simple-link protocol under each angle. As mentioned before, the computed set of paths from sensor nodes to the BS in simple-link forms an oriented forest with roots in the CHs.

The death of a node will cause a disconnection between its offspring and the BS. The situation will get even worse if the dead node is a CH. However, on one hand, nodes in the DRS are isolated from each other by configuring the network as a star topology. On the other hand, those dying nodes in RRP are also separated from others in reconfigurable routing. The death of a node in both DRS and RRP will not lead to a tremendous drop in connectivity, which helps prolong the network lifetime. In the comparison with RRP and DRS, DRS only applies direct communication while RRP tries to slow down the energy consumption through combining direct and multi-hop communications. Eventually, RRP achieves about 51.85%, 49.29%, 30.51%, and 15.94% of network lifetime extension over DRS under the scanning angles of $\frac{\pi }{6}$, $\frac{\pi }{4}$, $\frac{\pi }{3}$, and $\frac{\pi }{2}$, respectively.

### 5.3. Packet Delivery Ratio

The packet delivery ratio (PDR) is defined as the ratio of the total amount of packets transmitted at sensor nodes (*P_tx_*) to the total amount of packets received at the BS (*P_rx_*), shown as follows:

(14) $$\text{PDR}=\frac{\sum P_{rx}}{\sum P_{tx}}$$

**Figure 12.**
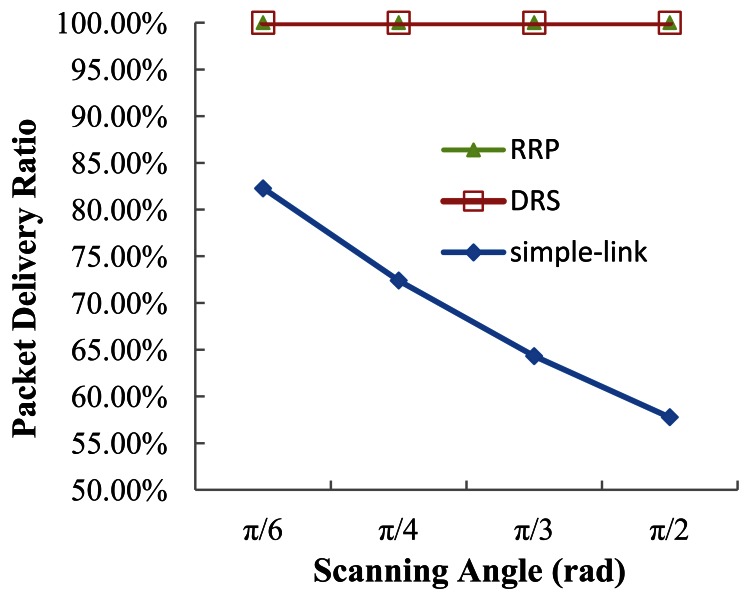
Packet delivery ratio *vs.* scanning angle.

The results of PDR under different scanning angles are given in Figure 12. Through maintaining the network connectivity, both DRS and RRP benefit from the relatively high PDR. However, this merit does not exist in the simple-link protocol that works without any reconfiguration.

**Figure 13.**
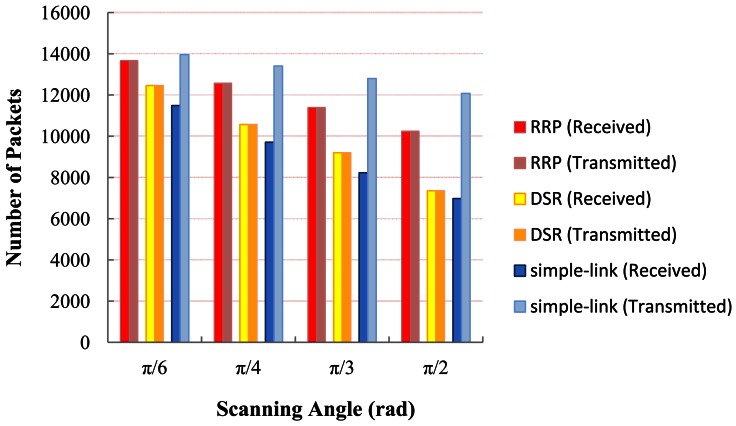
Number of delivered packets *vs.* scanning angle.

As in simple-link, the death of a node may probably trigger a great decrease of connectivity. The dead node is unable to continue forwarding data packets from its offspring even though they may still have sufficient energy to initiate data transmission. As a result, any data transmission originated from the dead node's offspring is meaningless and wastes energy. As expected, we observe that the PDR of the simple-link decreases with the growth of the scanning angle. That is because when the scanning angle increases, the probability of a node becoming the successor of other nodes increases. Once this node died of energy depletion, it might probably cause a large number of nodes to lose their contact with the BS, resulting in a decrease in PDR. Figure 13 gives the number of transmitted and received packets of each protocol under different scanning angles. The great difference between the number of transmitted and received packets in the simple-link protocol implies its inefficient data transmission, which is the result of poor maintenance of connectivity. Yet, both DRS and RRP indicate fairly well performance in this case compared to that of the simple-link.

**Figure 14.**
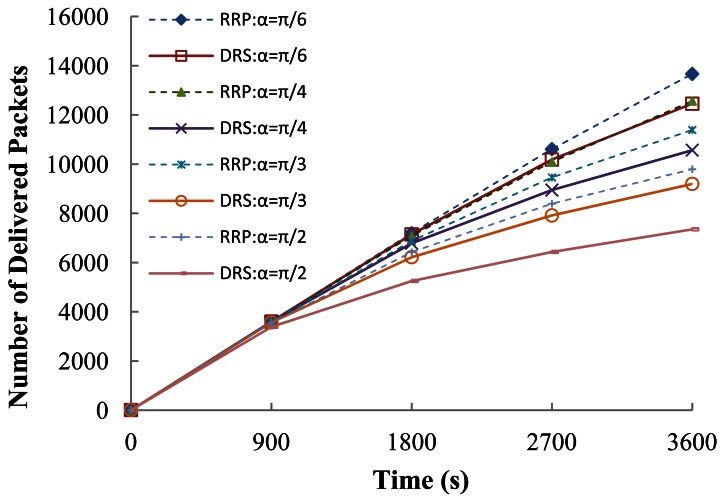
Number of delivered packets *vs.* time.

Note that within the same simulation time, the total number of delivered packets in RRP is larger than that of DRS. The reason is that nodes using RRP will die at a slower rate through the reconfiguration that combines direct and multi-hops communication, and they can transmit a higher number of packets. To better illustrate packet delivery in DRS and RRP, the results of delivered packets versus time are presented in Figure 14. RRP achieves better performance than DRS in each scanning angle. This is the merit brought by our proposed reconfiguration routing. Additionally, it is observed that the increment of delivered packets slows down as time goes by. This trend is expected because the number of transmitted packets per unit time will decrease with death of nodes.

### 5.4. The Number of Living Node

**Figure 15.**
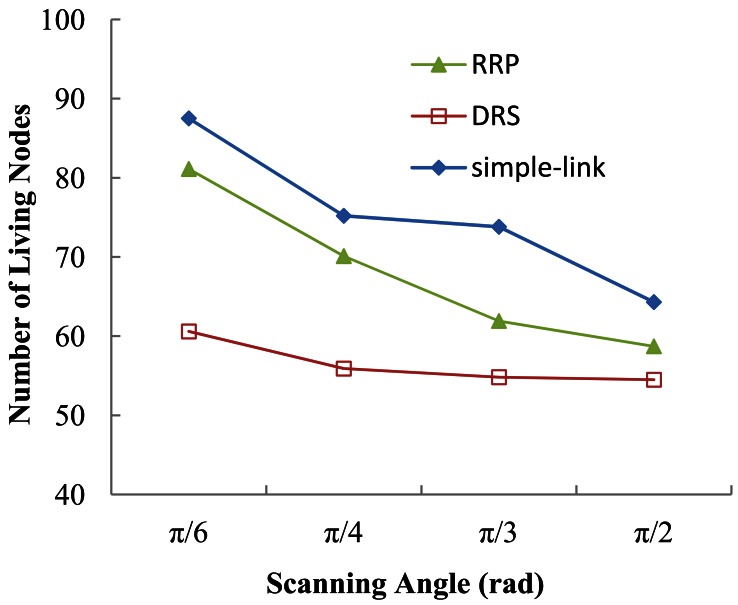
Number of living nodes *vs.* scanning angle.

The number of living nodes is also evaluated to examine the efficiency of each protocol. Figure 15 shows our simulation results. Under each scanning angle, the number of living nodes in the simple-link protocol is larger than that indicated by both RRP and DRS. Due to the special topology formed by simple-link, the death of a node is likely to bring about a dramatic decrease of connectivity. The successors of the dead node, which are supposed to relay packets from the dead node as well as the dead node's predecessors, may probably have a much smaller number of packets to transmit in this case. It helps decelerate their energy consumption rate, prolonging their lifetime. Nevertheless, both RRP and DRS aim to maintain the network connectivity for packet delivery. Though some nodes are out of energy during data transmission, other living nodes will continue transmitting packets to the BS without being affected by those dead ones. Thus, the number of transmission per node in RRP and DRS is greater than that in simple-link, eventually leading to a smaller value of living nodes. To a certain extent, the better performance achieved by the simple-link is a strong evidence of its inefficient data transmission. Note that within the same simulation time, as the scanning angle increases, the number of living nodes shows a decreasing trend in each protocol. This can be explained by the expended energy per transmission in the case of a larger scanning angle.

## 6. Discussion

The reconfiguration of RRP is achieved by dynamically exchanging message among the BS, the DN, ANs and MNs. This section discusses the impact of such a message exchange on the energy consumption during data transmission. This impact is shown by comparing the energy consumption of message exchange with that of data transmission.

Suppose the average hop from a node to its nearest CH or UCH is *hop_up_*, the size of the data packet is *Size_data_packet_*, then transmitting a data packet to the BS requires energy as follows:

(15) $$E_{\text{data}\_\text{transmission}}=(E_{AX}+E_{RX})\times {\text{hop}}_{up}\times \text{Siz}e_{\text{data}\_\text{packet}}+(E_{PX}\times \text{Siz}e_{\text{data}\_\text{packet}}+\alpha )$$

where *α* is a compensation factor. If the last node in the uplink path to the BS is an UCH, then the *α* is a positive number, and the (*E_PX_* × *Size_data_packet_* + *α*) equals to the energy required for the UCH transmitting the data packet to the BS with the active transmitter. However, *α* equals to 0 if the last node is a real CH which communicates with the BS using passive transmitter.

During data transmission, when a node's remaining energy reaches a threshold, it sends an SOS message to the BS. Let *Size_SOS_message_* be the size of SOS message, then the energy consumption for the SOS message to get to the BS, denoted by *E_SOS_*, is as follows:

(16) $$E_{\text{SOS}}=(E_{AX}+E_{RX})\times {\text{hop}}_{up}\times \text{Siz}e_{\text{SOS}\_\text{message}}+(E_{PX}\times \text{Siz}e_{\text{SOS}\_\text{messsage}}+\alpha )$$

After receiving the SOS message, the BS sends out reconfiguration messages directly to MNs, ANs, and the DN, taking advantage of their location information. Let *num_MN_* be the number of MNs, *num_AN_* be the number of ANs, and *Size_AN_*, *Size_MN_*, *Size_DN_* be the sizes of reconfiguration messages for ANs, MNs, and the DN respectively, then the energy consumption for nodes receiving the these messages, denoted by *E*_Re_*_conf_*, is:

(17) $$E_{\text{Reconf}}=E_{RX}\times \text{Siz}e_{AN}\times num_{AN}+E_{RX}\times \text{Siz}e_{MN}\times num_{MN}+E_{RX}\times \text{Siz}e_{DN}\times 1$$

The total energy needed for message exchange with respect to one reconfiguration is:

(18) $$E_{\text{message}\_\text{exchage}}=E_{\text{SOS}}+E_{\text{Re}\text{conf}}$$

Actually, the size of SOS message and reconfiguration messages are relatively small compared with the data packet. In our simulation, the SOS message is only 20 bits, including 4 bits for message type, and 16 bits for the DN's ID. The reconfiguration messages for DN, ANs, MNs are 20 bits, 132 bits and 36 bits respectively. The largest *num_AN_* and *num_MN_* in our simulation sets are 5 and 12 respectively. So if substituting these numbers for corresponding parameters in Equations (16–18), after simplification, we have:

(19) $$E_{\text{message}\_\text{exchage}}=20\times E_{AX}\times {\text{hop}}_{up}+(20\times {\text{hop}}_{up}+1112)\times E_{RX}+20\times E_{PX}+\alpha$$

Since the data packet is 2,048 bits, the energy consumption for delivering a data packet is:

(20) $$E_{\text{data}\_\text{transmission}}=2048\times E_{AX}\times {\text{hop}}_{up}+2048\times {\text{hop}}_{up}\times E_{RX}+2048\times E_{PX}+\alpha$$

According to Equations (19) and (20), it is intuitive to observe that *E_messagee_exchange_* is much smaller than *E_data_transmission_* as long as *hop_up_* is a positive number. And we know that this is always the case because the average number of hops never becomes negative. Besides, since each node is able to initiate a reconfiguration only once, the total number of reconfiguration initiation at most equals to the number of nodes in the network. Comparing to the tens of thousands of data packet transmission, the energy spent on message exchange for reconfiguration actually falls into acceptable range. In practice, it is expected to increase the size of data packet to balance the message exchange overhead.

## 7. Conclusions and Future Work

Due to the constraints of power and directionality, it is very essential and challenging to design communication protocols for FSOSNs. In this paper, we proposed RRP for FSOSNs to maintain network connectivity, guarantee packet delivery, and prolong network lifetime. In RRP, the data packets are first transmitted through the shortest paths. Then, a reconfiguration is initiated by the node whose residual energy has reached a threshold. Those nodes affected by this DN are classified into two types and reconfigured according to their types. The reconfiguration effectively prevents the DN from affecting the data transmission of other nodes, which contributes to improving the network performance. An energy model was setup to evaluate RRP. The simulation results indicate that RRP achieves better performance than the simple-link protocol and DRS in terms of connectivity, network lifetime, packet delivery ratio, and the number of living nodes.

In future, our work will focus on improving the scalability of the RRP. The RRP works well in a FSOSN with one BS compared to the simple-link protocol and DRS. But recent applications need to use multiple BSs in large scale WSNs in order to obtain balance network energy consumption and prolong network lifetime. The deployment of multiple BSs can increase the manageability of the network and reduce the energy dissipation at each node. Therefore, it is essential to extend the proposed RRP to support large scale FSOSNs with multiple BSs.

## Figures and Tables

**Table 1.** The routing table of each GS in Figure 2 after running the 2nd phase of RRP.

**Node**	**Next Hop Set**	**Cost**
*S_2_*	{*S_1_*}	2
*S_4_*	{*S_2_*, *S_7_*}	3
*S_5_*	{*S_4_*, *S_6_*}	4
*S_6_*	{*S_7_*}	3
*S_7_*	{*S_3_*}	2
*S_8_*	{S_9_}	3
S_9_	{*S_10_*}	2

**Table 2.** Denotations in AN's Reconfiguration.

**Denotation**	**Meaning**
{*CH*}	the set of living cluster heads
*S_AN_*	the AN of the dying node
*A_AN_*	the circle area that can be covered by the *S_AN_*
*Cost_i_*	the number of hops from *S_i_* to BS
{*Connected*}	the set of nodes satisfied connectivity requirement

**Table 3.** Energy Model.

**Operation**	**Energy Dissipation**
Active Transmitter (*ε*)	4 pJ/m^2^
Passive Transmitter (*E_CCR_*)	16 pJ/bit
Receiver (*E_R_*)	60 pJ/bit
